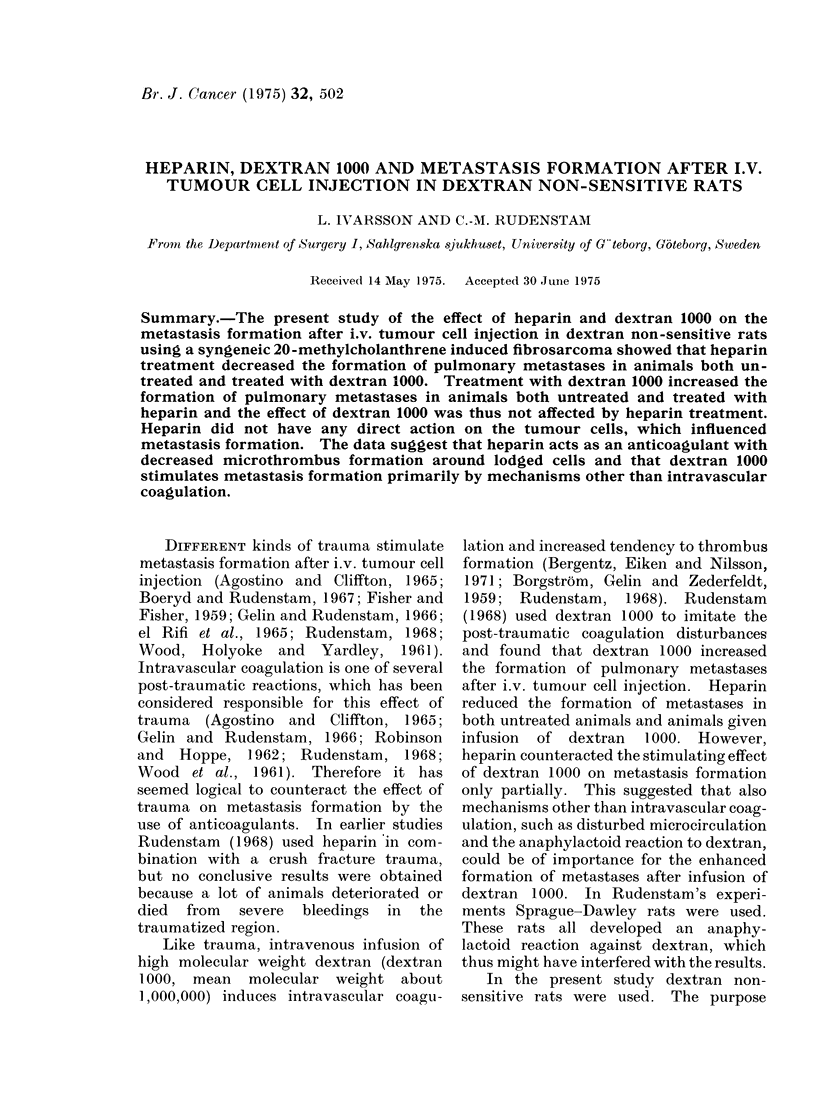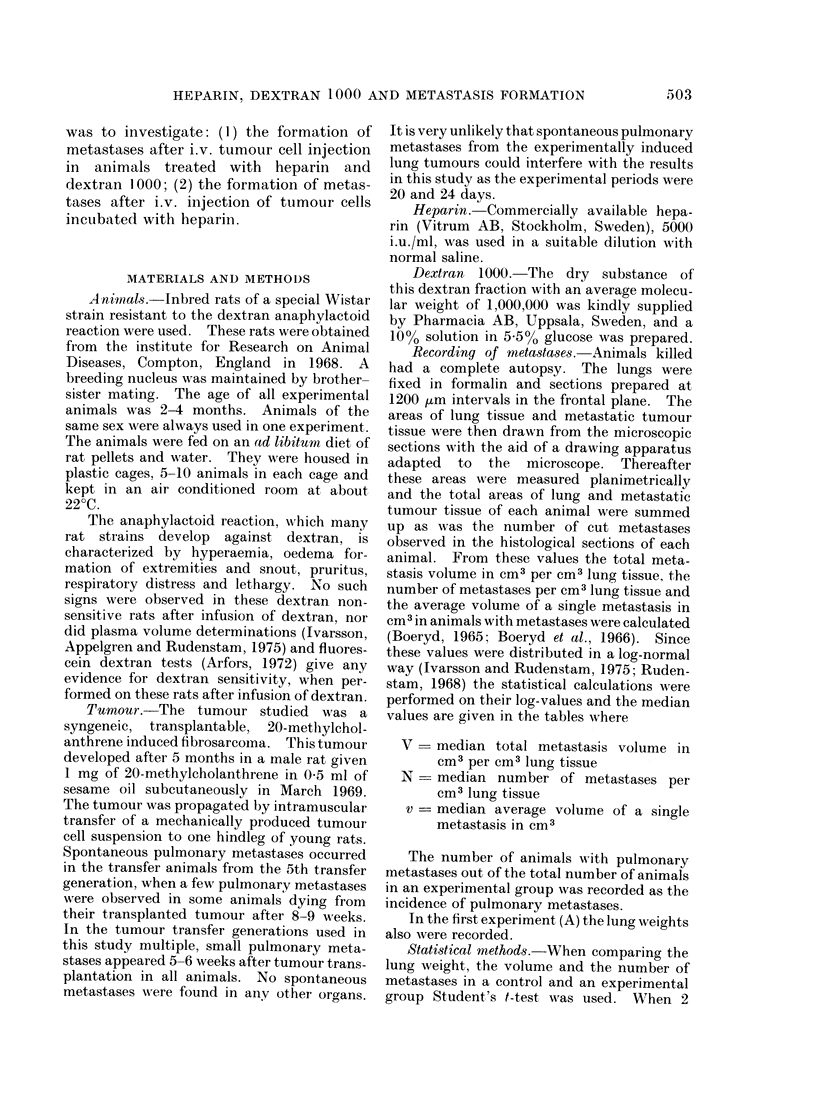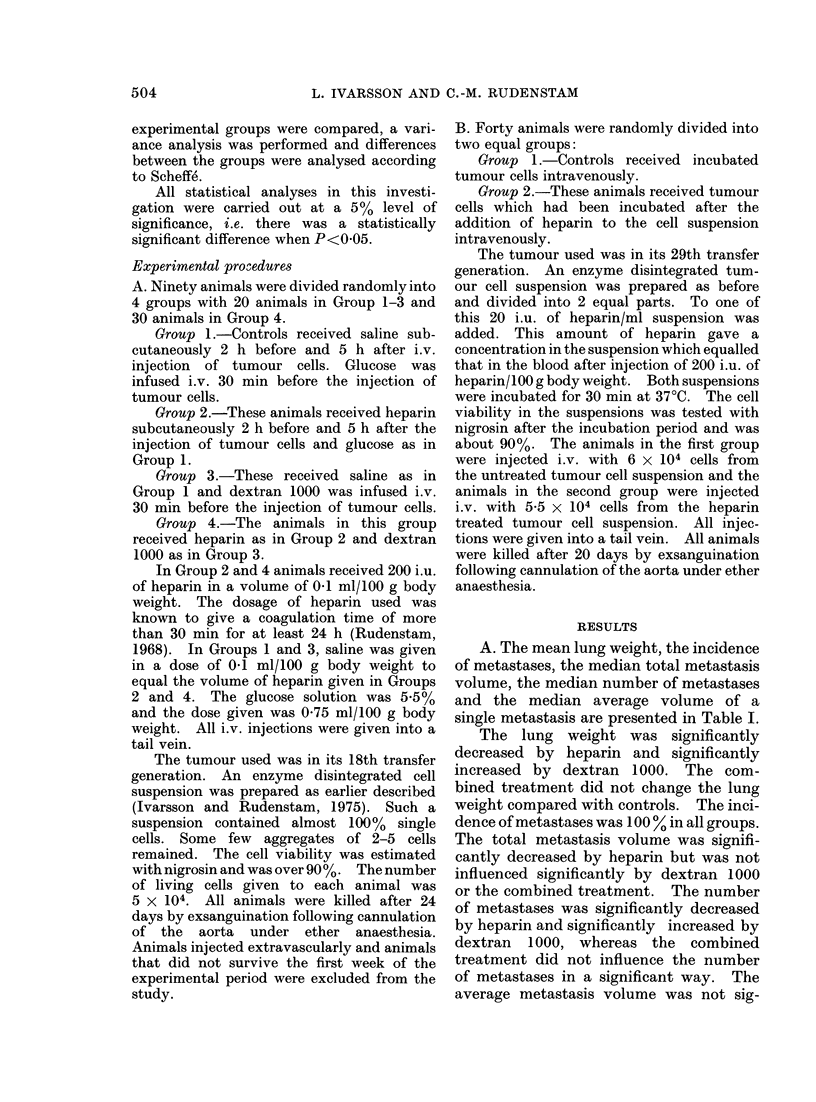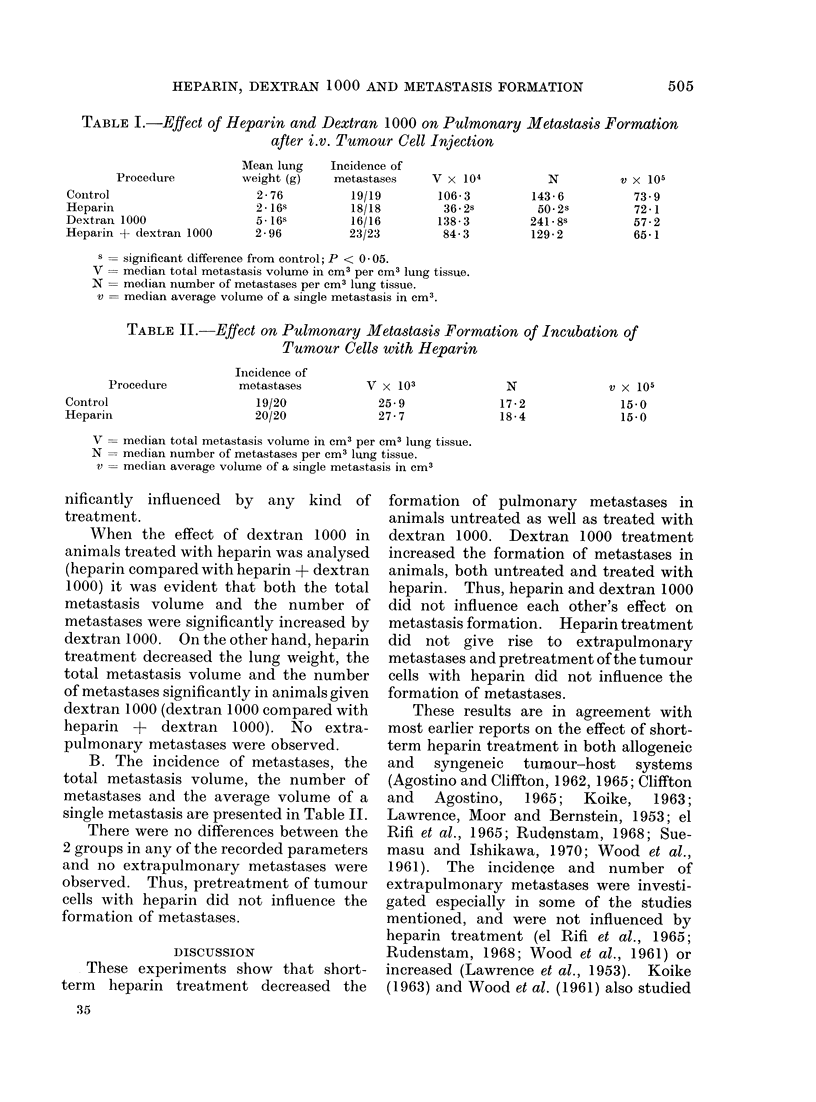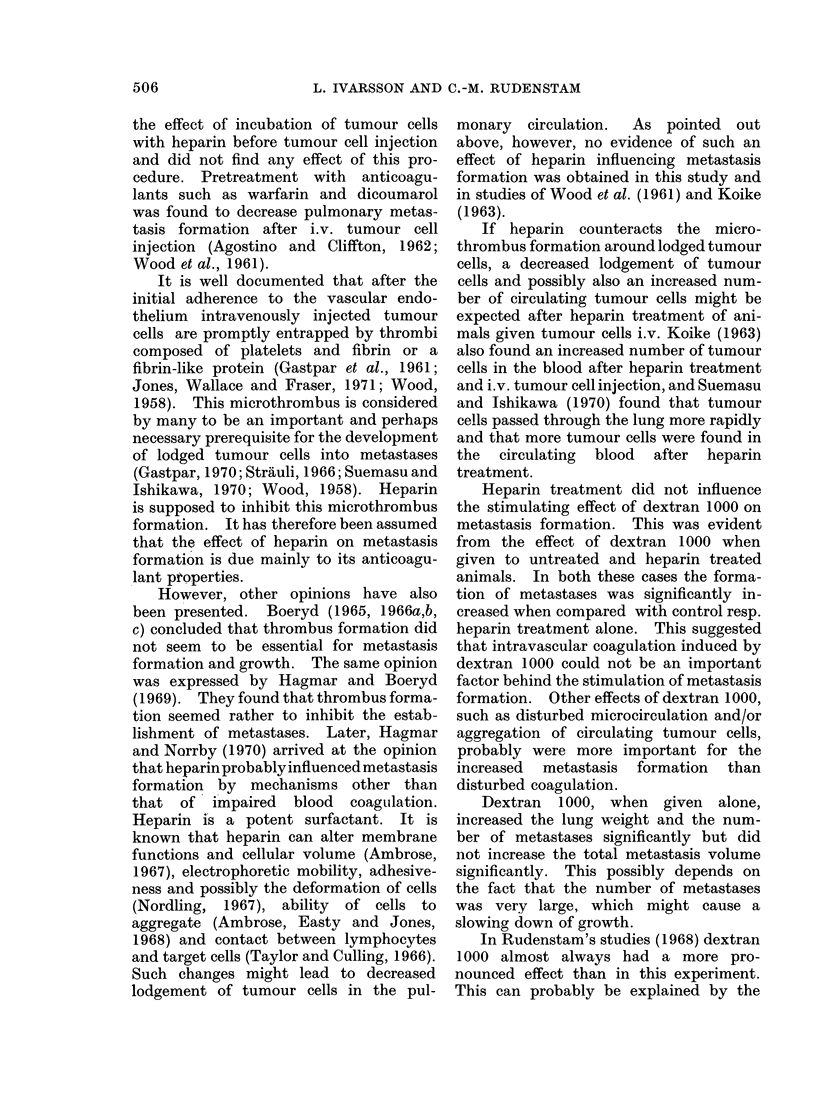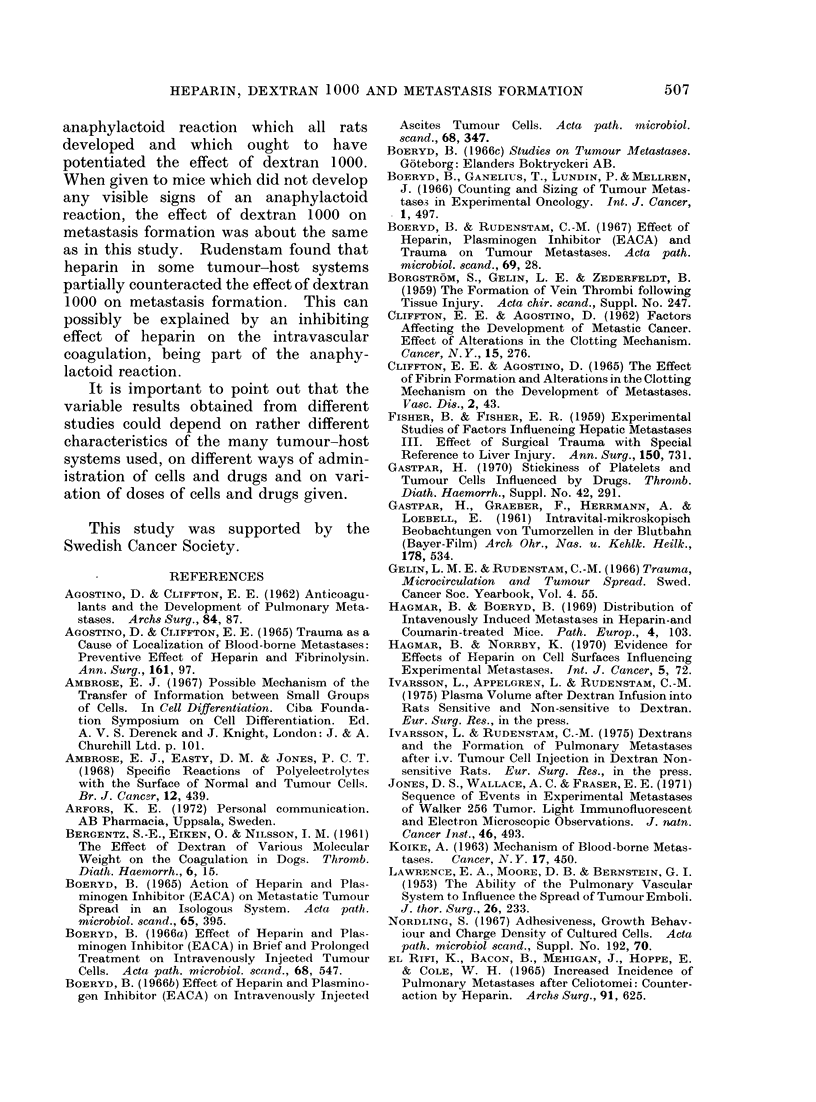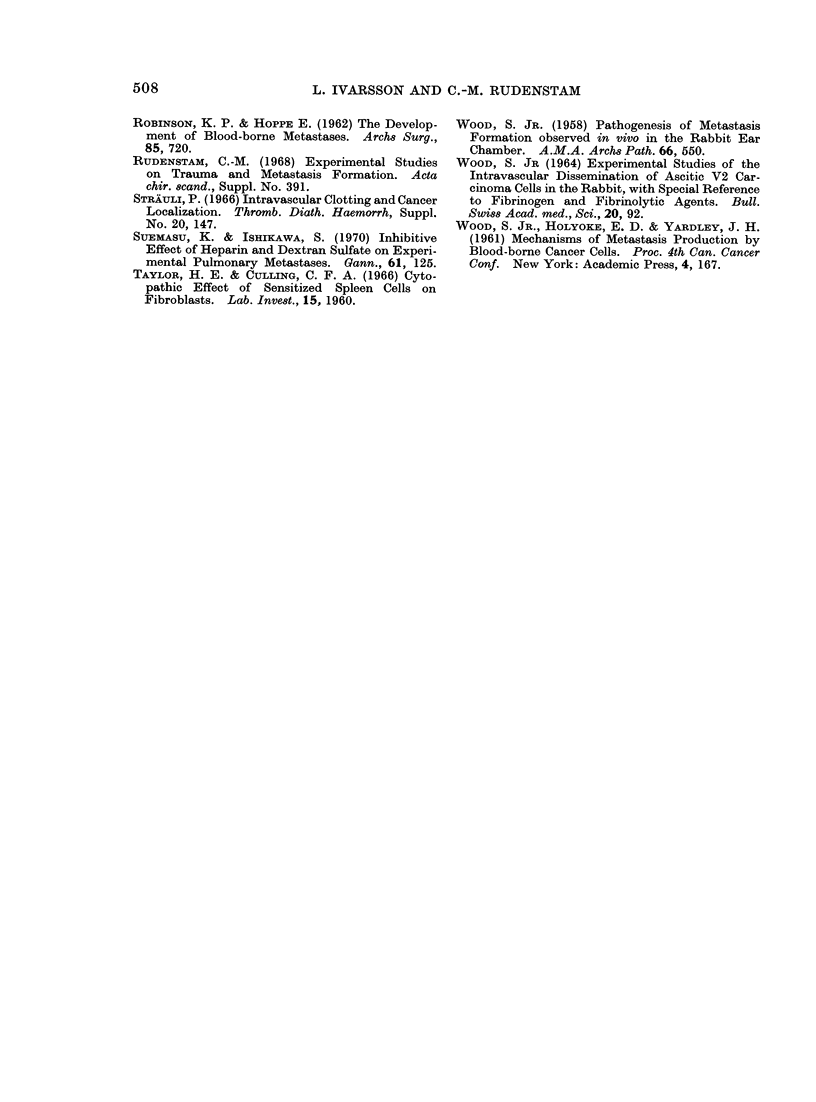# Heparin, dextran 1000 and metastasis formation after I.V. tumour cell injection in dextran non-sensitive rats.

**DOI:** 10.1038/bjc.1975.252

**Published:** 1975-10

**Authors:** L. Ivarsson, C. M. Rudenstam

## Abstract

The present study of the effect of heparin and dextran 1000 on the metastasis formation after i.v. tumour cell injection in dextran non-sensitive rats using a syngeneic 20-methylcholanthrene induced fibrosarcoma showed that heparin treatment decreased with formation of pulmonary metastases in animals both untreated and treated with dextran 1000. Treatment with dextran 1000 increased the formation of pulmonary metastases in animals both untreated and treated with heparin and the effect of dextran 1000 was thus not affected by heparin treatment. Heparin did not have any direct action on the tumour cells, which influenced metastasis formation. The data suggest that heparin acts as an anticoagulant with decreased microthrombus formation around lodged cells and that dextrax 1000 stimulates metastasis formation primarily by mechanisms other than intravascular coagulation.


*Br. J. Cancer* (1975) **32**, 502

### HEPARIN, DEXTRAN 1000 AND METASTASIS FORMATION AFTER I.V. TUMOUR CELL INJECTION IN DEXTRAN NON-SENSITIVE RATS

L. IVARSSON AND C.-M. RUDENSTAM

*From the Department of Surgery I, Sahlgrenska sjukhuset, University of G¨teborg, Göteborg, Sweden*

Received 14 May 1975. Accepted 30 June 1975

**Summary.—The present study of the effect of heparin and dextran 1000 on the metastasis formation after i.v. tumour cell injection in dextran non-sensitive rats using a syngeneic 20-methylcholanthrene induced fibrosarcoma showed that heparin treatment decreased the formation of pulmonary metastases in animals both untreated and treated with dextran 1000. Treatment with dextran 1000 increased the formation of pulmonary metastases in animals both untreated and treated with heparin and the effect of dextran 1000 was thus not affected by heparin treatment. Heparin did not have any direct action on the tumour cells, which influenced metastasis formation. The data suggest that heparin acts as an anticoagulant with decreased microthrombus formation around lodged cells and that dextran 1000 stimulates metastasis formation primarily by mechanisms other than intravascular coagulation.**

DIFFERENT kinds of trauma stimulate metastasis formation after i.v. tumour cell injection (Agostino and Cliffton, 1965; Boeryd and Rudenstam, 1967; Fisher and Fisher, 1959; Gelin and Rudenstam, 1966; el Rifi *et al.*, 1965; Rudenstam, 1968; Wood, Holyoke and Yardley, 1961). Intravascular coagulation is one of several post-traumatic reactions, which has been considered responsible for this effect of trauma (Agostino and Cliffton, 1965; Gelin and Rudenstam, 1966; Robinson and Hoppe, 1962; Rudenstam, 1968; Wood *et al.*, 1961). Therefore it has seemed logical to counteract the effect of trauma on metastasis formation by the use of anticoagulants. In earlier studies Rudenstam (1968) used heparin in combination with a crush fracture trauma, but no conclusive results were obtained because a lot of animals deteriorated or died from severe bleedings in the traumatized region.

Like trauma, intravenous infusion of high molecular weight dextran (dextran 1000, mean molecular weight about 1,000,000) induces intravascular coagulation and increased tendency to thrombus formation (Bergentz, Eiken and Nilsson, 1971; Borgström, Gelin and Zederfeldt, 1959; Rudenstam, 1968). Rudenstam (1968) used dextran 1000 to imitate the post-traumatic coagulation disturbances and found that dextran 1000 increased the formation of pulmonary metastases after i.v. tumour cell injection. Heparin reduced the formation of metastases in both untreated animals and animals given infusion of dextran 1000. However, heparin counteracted the stimulating effect of dextran 1000 on metastasis formation only partially. This suggested that also mechanisms other than intravascular coagulation, such as disturbed microcirculation and the anaphylactoid reaction to dextran, could be of importance for the enhanced formation of metastases after infusion of dextran 1000. In Rudenstam's experiments Sprague–Dawley rats were used. These rats all developed an anaphylactoid reaction against dextran, which thus might have interfered with the results.

In the present study dextran non-sensitive rats were used. The purpose was to investigate: (1) the formation of metastases after i.v. tumour cell injection in animals treated with heparin and dextran 1000; (2) the formation of metastases after i.v. injection of tumour cells incubated with heparin.

### MATERIALS AND METHODS

*Animals.*—Inbred rats of a special Wistar strain resistant to the dextran anaphylactoid reaction were used. These rats were obtained from the institute for Research on Animal Diseases, Compton, England in 1968. A breeding nucleus was maintained by brother-sister mating. The age of all experimental animals was 2–4 months. Animals of the same sex were always used in one experiment. The animals were fed on an *ad libitum* diet of rat pellets and water. They were housed in plastic cages, 5–10 animals in each cage and kept in an air conditioned room at about 22°C.

The anaphylactoid reaction, which many rat strains develop against dextran, is characterized by hyperaemia, oedema formation of extremities and snout, pruritus, respiratory distress and lethargy. No such signs were observed in these dextran non-sensitive rats after infusion of dextran, nor did plasma volume determinations (Ivarsson, Appelgren and Rudenstam, 1975) and fluorescein dextran tests (Arfors, 1972) give any evidence for dextran sensitivity, when performed on these rats after infusion of dextran.

*Tumour.*—The tumour studied was a syngeneic, transplantable, 20-methylcholanthrene induced fibrosarcoma. This tumour developed after 5 months in a male rat given 1 mg of 20-methylcholanthrene in 0·5 ml of sesame oil subcutaneously in March 1969. The tumour was propagated by intramuscular transfer of a mechanically produced tumour cell suspension to one hindleg of young rats. Spontaneous pulmonary metastases occurred in the transfer animals from the 5th transfer generation, when a few pulmonary metastases were observed in some animals dying from their transplanted tumour after 8–9 weeks. In the tumour transfer generations used in this study multiple, small pulmonary metastases appeared 5–6 weeks after tumour transplantation in all animals. No spontaneous metastases were found in any other organs. It is very unlikely that spontaneous pulmonary metastases from the experimentally induced lung tumours could interfere with the results in this study as the experimental periods were 20 and 24 days.

*Heparin.*—Commercially available heparin (Vitrum AB, Stockholm, Sweden), 5000 i.u./ml, was used in a suitable dilution with normal saline.

*Dextran* 1000.—The dry substance of this dextran fraction with an average molecular weight of 1,000,000 was kindly supplied by Pharmacia AB, Uppsala, Sweden, and a 10% solution in 5·5% glucose was prepared.

*Recording of metastases.*—Animals killed had a complete autopsy. The lungs were fixed in formalin and sections prepared at 1200 μm intervals in the frontal plane. The areas of lung tissue and metastatic tumour tissue were then drawn from the microscopic sections with the aid of a drawing apparatus adapted to the microscope. Thereafter these areas were measured planimetrically and the total areas of lung and metastatic tumour tissue of each animal were summed up as was the number of cut metastases observed in the histological sections of each animal. From these values the total metastasis volume in $cm^3$ per $cm^3$ lung tissue, the number of metastases per $cm^3$ lung tissue and the average volume of a single metastasis in $cm^3$ in animals with metastases were calculated (Boeryd, 1965; Boeryd *et al.*, 1966). Since these values were distributed in a log-normal way (Ivarsson and Rudenstam, 1975; Rudenstam, 1968) the statistical calculations were performed on their log-values and the median values are given in the tables where

V = median total metastasis volume in $cm^3$ per $cm^3$ lung tissue
N = median number of metastases per $cm^3$ lung tissue
$v$ = median average volume of a single metastasis in $cm^3$

The number of animals with pulmonary metastases out of the total number of animals in an experimental group was recorded as the incidence of pulmonary metastases.

In the first experiment (A) the lung weights also were recorded.

*Statistical methods.*—When comparing the lung weight, the volume and the number of metastases in a control and an experimental group Student's *t*-test was used. When 2

experimental groups were compared, a variance analysis was performed and differences between the groups were analysed according to Scheffé.

All statistical analyses in this investigation were carried out at a 5% level of significance, *i.e.* there was a statistically significant difference when $P<0{\cdot}05$.

*Experimental procedures*

A. Ninety animals were divided randomly into 4 groups with 20 animals in Group 1–3 and 30 animals in Group 4.

*Group* 1.—Controls received saline subcutaneously 2 h before and 5 h after i.v. injection of tumour cells. Glucose was infused i.v. 30 min before the injection of tumour cells.

*Group* 2.—These animals received heparin subcutaneously 2 h before and 5 h after the injection of tumour cells and glucose as in Group 1.

*Group* 3.—These received saline as in Group 1 and dextran 1000 was infused i.v. 30 min before the injection of tumour cells.

*Group* 4.—The animals in this group received heparin as in Group 2 and dextran 1000 as in Group 3.

In Group 2 and 4 animals received 200 i.u. of heparin in a volume of 0·1 ml/100 g body weight. The dosage of heparin used was known to give a coagulation time of more than 30 min for at least 24 h (Rudenstam, 1968). In Groups 1 and 3, saline was given in a dose of 0·1 ml/100 g body weight to equal the volume of heparin given in Groups 2 and 4. The glucose solution was 5·5% and the dose given was 0·75 ml/100 g body weight. All i.v. injections were given into a tail vein.

The tumour used was in its 18th transfer generation. An enzyme disintegrated cell suspension was prepared as earlier described (Ivarsson and Rudenstam, 1975). Such a suspension contained almost 100% single cells. Some few aggregates of 2–5 cells remained. The cell viability was estimated with nigrosin and was over 90%. The number of living cells given to each animal was $5 \times 10^4$. All animals were killed after 24 days by exsanguination following cannulation of the aorta under ether anaesthesia. Animals injected extravascularly and animals that did not survive the first week of the experimental period were excluded from the study.

B. Forty animals were randomly divided into two equal groups:

*Group* 1.—Controls received incubated tumour cells intravenously.

*Group* 2.—These animals received tumour cells which had been incubated after the addition of heparin to the cell suspension intravenously.

The tumour used was in its 29th transfer generation. An enzyme disintegrated tumour cell suspension was prepared as before and divided into 2 equal parts. To one of this 20 i.u. of heparin/ml suspension was added. This amount of heparin gave a concentration in the suspension which equalled that in the blood after injection of 200 i.u. of heparin/100 g body weight. Both suspensions were incubated for 30 min at 37°C. The cell viability in the suspensions was tested with nigrosin after the incubation period and was about 90%. The animals in the first group were injected i.v. with $6 \times 10^4$ cells from the untreated tumour cell suspension and the animals in the second group were injected i.v. with $5{\cdot}5 \times 10^4$ cells from the heparin treated tumour cell suspension. All injections were given into a tail vein. All animals were killed after 20 days by exsanguination following cannulation of the aorta under ether anaesthesia.

### RESULTS

A. The mean lung weight, the incidence of metastases, the median total metastasis volume, the median number of metastases and the median average volume of a single metastasis are presented in Table I.

The lung weight was significantly decreased by heparin and significantly increased by dextran 1000. The combined treatment did not change the lung weight compared with controls. The incidence of metastases was 100% in all groups. The total metastasis volume was significantly decreased by heparin but was not influenced significantly by dextran 1000 or the combined treatment. The number of metastases was significantly decreased by heparin and significantly increased by dextran 1000, whereas the combined treatment did not influence the number of metastases in a significant way. The average metastasis volume was not sig-

TABLE I.—*Effect of Heparin and Dextran* 1000 *on Pulmonary Metastasis Formation after i.v. Tumour Cell Injection*

| Procedure | Mean lung weight (g) | Incidence of metastases | $V \times 10^4$ | N | $v \times 10^5$ |
|---|---|---|---|---|---|
| Control | 2·76 | 19/19 | 106·3 | 143·6 | 73·9 |
| Heparin | $2\cdot16^s$ | 18/18 | $36\cdot2^s$ | $50\cdot2^s$ | 72·1 |
| Dextran 1000 | $5\cdot16^s$ | 16/16 | 138·3 | $241\cdot8^s$ | 57·2 |
| Heparin + dextran 1000 | 2·96 | 23/23 | 84·3 | 129·2 | 65·1 |

$^s$ = significant difference from control; $P < 0\cdot05$.
V = median total metastasis volume in $cm^3$ per $cm^3$ lung tissue.
N = median number of metastases per $cm^3$ lung tissue.
$v$ = median average volume of a single metastasis in $cm^3$.

TABLE II.—*Effect on Pulmonary Metastasis Formation of Incubation of Tumour Cells with Heparin*

| Procedure | Incidence of metastases | $V \times 10^3$ | N | $v \times 10^5$ |
|---|---|---|---|---|
| Control | 19/20 | 25·9 | 17·2 | 15·0 |
| Heparin | 20/20 | 27·7 | 18·4 | 15·0 |

V = median total metastasis volume in $cm^3$ per $cm^3$ lung tissue.
N = median number of metastases per $cm^3$ lung tissue.
$v$ = median average volume of a single metastasis in $cm^3$

nificantly influenced by any kind of treatment.

When the effect of dextran 1000 in animals treated with heparin was analysed (heparin compared with heparin + dextran 1000) it was evident that both the total metastasis volume and the number of metastases were significantly increased by dextran 1000. On the other hand, heparin treatment decreased the lung weight, the total metastasis volume and the number of metastases significantly in animals given dextran 1000 (dextran 1000 compared with heparin + dextran 1000). No extrapulmonary metastases were observed.

B. The incidence of metastases, the total metastasis volume, the number of metastases and the average volume of a single metastasis are presented in Table II.

There were no differences between the 2 groups in any of the recorded parameters and no extrapulmonary metastases were observed. Thus, pretreatment of tumour cells with heparin did not influence the formation of metastases.

### DISCUSSION

These experiments show that short-term heparin treatment decreased the formation of pulmonary metastases in animals untreated as well as treated with dextran 1000. Dextran 1000 treatment increased the formation of metastases in animals, both untreated and treated with heparin. Thus, heparin and dextran 1000 did not influence each other's effect on metastasis formation. Heparin treatment did not give rise to extrapulmonary metastases and pretreatment of the tumour cells with heparin did not influence the formation of metastases.

These results are in agreement with most earlier reports on the effect of short-term heparin treatment in both allogeneic and syngeneic tumour–host systems (Agostino and Cliffton, 1962, 1965; Cliffton and Agostino, 1965; Koike, 1963; Lawrence, Moor and Bernstein, 1953; el Rifi *et al.*, 1965; Rudenstam, 1968; Suemasu and Ishikawa, 1970; Wood *et al.*, 1961). The incidence and number of extrapulmonary metastases were investigated especially in some of the studies mentioned, and were not influenced by heparin treatment (el Rifi *et al.*, 1965; Rudenstam, 1968; Wood *et al.*, 1961) or increased (Lawrence *et al.*, 1953). Koike (1963) and Wood *et al.* (1961) also studied the effect of incubation of tumour cells with heparin before tumour cell injection and did not find any effect of this procedure. Pretreatment with anticoagulants such as warfarin and dicoumarol was found to decrease pulmonary metastasis formation after i.v. tumour cell injection (Agostino and Cliffton, 1962; Wood *et al.*, 1961).

It is well documented that after the initial adherence to the vascular endothelium intravenously injected tumour cells are promptly entrapped by thrombi composed of platelets and fibrin or a fibrin-like protein (Gastpar *et al.*, 1961; Jones, Wallace and Fraser, 1971; Wood, 1958). This microthrombus is considered by many to be an important and perhaps necessary prerequisite for the development of lodged tumour cells into metastases (Gastpar, 1970; Sträuli, 1966; Suemasu and Ishikawa, 1970; Wood, 1958). Heparin is supposed to inhibit this microthrombus formation. It has therefore been assumed that the effect of heparin on metastasis formation is due mainly to its anticoagulant properties.

However, other opinions have also been presented. Boeryd (1965, 1966*a*,*b*, *c*) concluded that thrombus formation did not seem to be essential for metastasis formation and growth. The same opinion was expressed by Hagmar and Boeryd (1969). They found that thrombus formation seemed rather to inhibit the establishment of metastases. Later, Hagmar and Norrby (1970) arrived at the opinion that heparin probably influenced metastasis formation by mechanisms other than that of impaired blood coagulation. Heparin is a potent surfactant. It is known that heparin can alter membrane functions and cellular volume (Ambrose, 1967), electrophoretic mobility, adhesiveness and possibly the deformation of cells (Nordling, 1967), ability of cells to aggregate (Ambrose, Easty and Jones, 1968) and contact between lymphocytes and target cells (Taylor and Culling, 1966). Such changes might lead to decreased lodgement of tumour cells in the pulmonary circulation. As pointed out above, however, no evidence of such an effect of heparin influencing metastasis formation was obtained in this study and in studies of Wood *et al.* (1961) and Koike (1963).

If heparin counteracts the microthrombus formation around lodged tumour cells, a decreased lodgement of tumour cells and possibly also an increased number of circulating tumour cells might be expected after heparin treatment of animals given tumour cells i.v. Koike (1963) also found an increased number of tumour cells in the blood after heparin treatment and i.v. tumour cell injection, and Suemasu and Ishikawa (1970) found that tumour cells passed through the lung more rapidly and that more tumour cells were found in the circulating blood after heparin treatment.

Heparin treatment did not influence the stimulating effect of dextran 1000 on metastasis formation. This was evident from the effect of dextran 1000 when given to untreated and heparin treated animals. In both these cases the formation of metastases was significantly increased when compared with control resp. heparin treatment alone. This suggested that intravascular coagulation induced by dextran 1000 could not be an important factor behind the stimulation of metastasis formation. Other effects of dextran 1000, such as disturbed microcirculation and/or aggregation of circulating tumour cells, probably were more important for the increased metastasis formation than disturbed coagulation.

Dextran 1000, when given alone, increased the lung weight and the number of metastases significantly but did not increase the total metastasis volume significantly. This possibly depends on the fact that the number of metastases was very large, which might cause a slowing down of growth.

In Rudenstam's studies (1968) dextran 1000 almost always had a more pronounced effect than in this experiment. This can probably be explained by the anaphylactoid reaction which all rats developed and which ought to have potentiated the effect of dextran 1000. When given to mice which did not develop any visible signs of an anaphylactoid reaction, the effect of dextran 1000 on metastasis formation was about the same as in this study. Rudenstam found that heparin in some tumour–host systems partially counteracted the effect of dextran 1000 on metastasis formation. This can possibly be explained by an inhibiting effect of heparin on the intravascular coagulation, being part of the anaphylactoid reaction.

It is important to point out that the variable results obtained from different studies could depend on rather different characteristics of the many tumour–host systems used, on different ways of administration of cells and drugs and on variation of doses of cells and drugs given.

This study was supported by the Swedish Cancer Society.

### REFERENCES

AGOSTINO, D. & CLIFFTON, E. E. (1962) Anticoagulants and the Development of Pulmonary Metastases. *Archs Surg.*, **84**, 87.

AGOSTINO, D. & CLIFFTON, E. E. (1965) Trauma as a Cause of Localization of Blood-borne Metastases: Preventive Effect of Heparin and Fibrinolysin. *Ann. Surg.*, **161**, 97.

AMBROSE, E. J. (1967) Possible Mechanism of the Transfer of Information between Small Groups of Cells. In *Cell Differentiation.* Ciba Foundation Symposium on Cell Differentiation. Ed. A. V. S. Derenck and J. Knight, London: J. & A. Churchill Ltd. p. 101.

AMBROSE, E. J., EASTY, D. M. & JONES, P. C. T. (1968) Specific Reactions of Polyelectrolytes with the Surface of Normal and Tumour Cells. *Br. J. Cancer*, **12**, 439.

ARFORS, K. E. (1972) Personal communication. AB Pharmacia, Uppsala, Sweden.

BERGENTZ, S.-E., EIKEN, O. & NILSSON, I. M. (1961) The Effect of Dextran of Various Molecular Weight on the Coagulation in Dogs. *Thromb. Diath. Haemorrh.*, **6**, 15.

BOERYD, B. (1965) Action of Heparin and Plasminogen Inhibitor (EACA) on Metastatic Tumour Spread in an Isologous System. *Acta path. microbiol. scand.*, **65**, 395.

BOERYD, B. (1966*a*) Effect of Heparin and Plasminogen Inhibitor (EACA) in Brief and Prolonged Treatment on Intravenously Injected Tumour Cells. *Acta path. microbiol. scand.*, **68**, 547.

BOERYD, B. (1966*b*) Effect of Heparin and Plasminogen Inhibitor (EACA) on Intravenously Injected Ascites Tumour Cells. *Acta path. microbiol. scand.*, **68**, **347.**

BOERYD, B. (1966*c*) *Studies on Tumour Metastases.* Göteborg: Elanders Boktryckeri AB.

BOERYD, B., GANELIUS, T., LUNDIN, P. & MELLREN, J. (1966) Counting and Sizing of Tumour Metastases in Experimental Oncology. *Int. J. Cancer*, **1**, 497.

BOERYD, B. & RUDENSTAM, C.-M. (1967) Effect of Heparin, Plasminogen Inhibitor (EACA) and Trauma on Tumour Metastases. *Acta path. microbiol. scand.*, **69**, 28.

BORGSTRÖM, S., GELIN, L. E. & ZEDERFELDT, B. (1959) The Formation of Vein Thrombi following Tissue Injury. *Acta chir. scand.*, Suppl. No. 247.

CLIFFTON, E. E. & AGOSTINO, D. (1962) Factors Affecting the Development of Metastic Cancer. Effect of Alterations in the Clotting Mechanism. *Cancer, N.Y.*, **15**, 276.

CLIFFTON, E. E. & AGOSTINO, D. (1965) The Effect of Fibrin Formation and Alterations in the Clotting Mechanism on the Development of Metastases. *Vasc. Dis.*, **2**, 43.

FISHER, B. & FISHER, E. R. (1959) Experimental Studies of Factors Influencing Hepatic Metastases III. Effect of Surgical Trauma with Special Reference to Liver Injury. *Ann. Surg.*, **150**, 731.

GASTPAR, H. (1970) Stickiness of Platelets and Tumour Cells Influenced by Drugs. *Thromb. Diath. Haemorrh.*, Suppl. No. 42, 291.

GASTPAR, H., GRAEBER, F., HERRMANN, A. & LOEBELL, E. (1961) Intravital-mikroskopisch Beobachtungen von Tumorzellen in der Blutbahn (Bayer-Film) *Arch Ohr., Nas. u. Kehlk. Heilk.*, **178**, 534.

GELIN, L. M. E. & RUDENSTAM, C.-M. (1966) *Trauma, Microcirculation and Tumour Spread.* Swed. Cancer Soc. Yearbook, Vol. 4. 55.

HAGMAR, B. & BOERYD, B. (1969) Distribution of Intavenously Induced Metastases in Heparin-and Coumarin-treated Mice. *Path. Europ.*, **4**, 103.

HAGMAR, B. & NORRBY, K. (1970) Evidence for Effects of Heparin on Cell Surfaces Influencing Experimental Metastases. *Int. J. Cancer*, **5**, 72.

IVARSSON, L., APPELGREN, L. & RUDENSTAM, C.-M. (1975) Plasma Volume after Dextran Infusion into Rats Sensitive and Non-sensitive to Dextran. *Eur. Surg. Res.*, in the press.

IVARSSON, L. & RUDENSTAM, C.-M. (1975) Dextrans and the Formation of Pulmonary Metastases after i.v. Tumour Cell Injection in Dextran Non-sensitive Rats. *Eur. Surg. Res.*, in the press.

JONES, D. S., WALLACE, A. C. & FRASER, E. E. (1971) Sequence of Events in Experimental Metastases of Walker 256 Tumor. Light Immunofluorescent and Electron Microscopic Observations. *J. natn. Cancer Inst.*, **46**, 493.

KOIKE, A. (1963) Mechanism of Blood-borne Metastases. *Cancer, N.Y.* **17**, 450.

LAWRENCE, E. A., MOORE, D. B. & BERNSTEIN, G. I. (1953) The Ability of the Pulmonary Vascular System to Influence the Spread of Tumour Emboli. *J. thor. Surg.*, **26**, 233.

NORDLING, S. (1967) Adhesiveness, Growth Behaviour and Charge Density of Cultured Cells. *Acta path. microbiol scand.*, Suppl. No. 192, **70.**

EL RIFI, K., BACON, B., MEHIGAN, J., HOPPE, E. & COLE, W. H. (1965) Increased Incidence of Pulmonary Metastases after Celiotomei: Counteraction by Heparin. *Archs Surg.*, **91**, 625.

ROBINSON, K. P. & HOPPE E. (1962) The Development of Blood-borne Metastases. *Archs Surg.*, **85**, 720.

RUDENSTAM, C.-M. (1968) Experimental Studies on Trauma and Metastasis Formation. *Acta chir. scand.*, Suppl. No. 391.

STRÄULI, P. (1966) Intravascular Clotting and Cancer Localization. *Thromb. Diath. Haemorrh*, Suppl. No. 20, 147.

SUEMASU, K. & ISHIKAWA, S. (1970) Inhibitive Effect of Heparin and Dextran Sulfate on Experimental Pulmonary Metastases. *Gann.*, **61**, 125.

TAYLOR, H. E. & CULLING, C. F. A. (1966) Cytopathic Effect of Sensitized Spleen Cells on Fibroblasts. *Lab. Invest.*, **15**, 1960.

WOOD, S. JR. (1958) Pathogenesis of Metastasis Formation observed *in vivo* in the Rabbit Ear Chamber. *A.M.A. Archs Path.* **66**, 550.

WOOD, S. JR (1964) Experimental Studies of the Intravascular Dissemination of Ascitic V2 Carcinoma Cells in the Rabbit, with Special Reference to Fibrinogen and Fibrinolytic Agents. *Bull. Swiss Acad. med., Sci.*, **20**, 92.

WOOD, S. JR., HOLYOKE, E. D. & YARDLEY, J. H. (1961) Mechanisms of Metastasis Production by Blood-borne Cancer Cells. *Proc. 4th Can. Cancer Conf.* New York: Academic Press, **4**, 167.